# Chicken jejunal microbiota improves growth performance by mitigating intestinal inflammation

**DOI:** 10.1186/s40168-022-01299-8

**Published:** 2022-07-15

**Authors:** Xiaolong Zhang, Muhammad Akhtar, Yan Chen, Ziyu Ma, Yuyun Liang, Deshi Shi, Ranran Cheng, Lei Cui, Yafang Hu, Abdallah A. Nafady, Abdur Rahman Ansari, El-Sayed M. Abdel-Kafy, Huazhen Liu

**Affiliations:** 1 Department of Basic Veterinary Medicine, College of Animal Science and Veterinary Medicine, Huazhong Agricultural University, Wuhan, 430070 Hubei China; 2 Department of Preventive Veterinary Medicine, College of Animal Science and Veterinary Medicine, Huazhong Agricultural University, Wuhan, 430070 Hubei China; 3 Section of Anatomy and Histology, Department of Basic Sciences, College of Veterinary and Animal Sciences (CVAS) Jhang, University of Veterinary and Animal Sciences (UVAS), Lahore, Pakistan; 4 Animal Production Research Institute (APRI), Agricultural Research Center (ARC), Ministry of Agriculture, Giza, Egypt

**Keywords:** Chicken, Jejunal microbiota, Intestinal inflammation, Growth performance, Fecal microbiota transplantation

## Abstract

**Background:**

Intestinal inflammation is prevalent in chicken, which results in decreased growth performance and considerable economic losses. Accumulated findings established the close relationship between gut microbiota and chicken growth performance. However, whether gut microbiota impacts chicken growth performance by lessening intestinal inflammation remains elusive.

**Results:**

Seven-weeks-old male and female chickens with the highest or lowest body weights were significantly different in breast and leg muscle indices and average cross-sectional area of muscle cells. 16S rRNA gene sequencing indicated Gram-positive bacteria, such as *Lactobacilli*, were the predominant species in high body weight chickens. Conversely, Gram-negative bacteria, such as *Comamonas*, *Acinetobacter*, *Brucella*, *Escherichia-Shigella*, *Thermus*, *Undibacterium*, and *Allorhizobium-Neorhizobium-Pararhizobium-Rhizobium* were significantly abundant in low body weight chickens. Serum lipopolysaccharide (LPS) level was significantly higher in low body weight chickens (101.58 ± 5.78 ng/mL) compared with high body weight chickens (85.12 ± 4.79 ng/mL). The expression of TLR4, NF-κB, MyD88, and related inflammatory cytokines in the jejunum was significantly upregulated in low body weight chickens, which led to the damage of gut barrier integrity. Furthermore, transferring fecal microbiota from adult chickens with high body weight into 1-day-old chicks reshaped the jejunal microbiota, mitigated inflammatory response, and improved chicken growth performance.

**Conclusions:**

Our findings suggested that jejunal microbiota could affect chicken growth performance by mitigating intestinal inflammation.

Video Abstract

**Supplementary Information:**

The online version contains supplementary material available at 10.1186/s40168-022-01299-8.

## Introduction

Intestinal inflammation imposes several threats to the chickens, including decreased feed intake, abnormal food digestion and absorption, and low meat production, resulting in reduced growth performance [1]. Intestinal inflammation also impairs gut homeostasis [2]. Disruption of gut homeostasis is associated with several pathological states that facilitate to flourish pathogens, causing multiple complications in chickens [3]. Infectious agents may damage the intestinal mucosa, initiate inflammation, disrupt gastrointestinal tract physiological mechanisms, and cause infectious and inflammatory diseases [4]. Over the years, antibiotics have been traditionally used as growth promoters in chickens. However, excessive and indiscriminate usage emerges antibiotic resistant strains, i.e., *Clostridium perfringens*, *Escherichia coli*, *Salmonella enterica*, and *Campylobacter* spp., which can be transmitted to humans and threaten public health and food safety [5, 6]. Rising concerns of antibiotic resistance have urged many countries to ban antibiotic growth promoters in food animal production [7]. Hence, focusing on intestinal physiology is a timely alternative approach for chicken production.

It has been established that gut microbiota consists of a complex consortium of microbial communities and colonizes in the chicken gastrointestinal tract, and the highest and dynamic bacterial diversity is observed in the cecum [8, 9]. Accumulated pieces of evidence from the chicken cecum have indicated the importance of gut microbiota in improving feed digestion, nutrient absorption, host defence, and immune response [10–12]. A stable microbiota in the host gut prevents pathogenic colonization and facilitates the clearance of infectious agents, thus helps improve the growth performance [13]. In the recent decade, the application of fecal microbiota transplantation (FMT) or probiotic supplementation has been emerging as a potential therapeutic strategy and an intervention approach to reconstitute intestinal flora, reduce the inflammatory response, and promote growth and development [14–18]. For instance, transferring fecal microbiota from healthy adult chickens could influence early colonization of gut microbiota and might have long-term consequences on the host-microbe interaction and development of the recipient chickens [19]. FMT could be useful for improving body weight gain, pathogens tolerance in broilers [20], and growth performance in calves [21]. Glendinning et al. [22] successfully transplanted the cecal microbiota from Roslin broilers to different chicken breeds in the first week of life and found increased microbiota richness and diversity in the recipients. FMT also improved the intestinal morphology in broiler by increasing the thickness of its serous membrane and muscle layers [20]. Due to the jejunum’s unique features, i.e., efficient nutrients absorption via the largest surface area [23], and efficient nutrient translocation via active vascular system of villi [24], nutrient absorption/intake mainly occurs in the jejunum. The jejunal histomorphology is a good indicator for digestive/absorptive ability [25]. It has also been reported that jejunal microbiota considerably contributes to the nutrient uptake and utilization in broilers [26]. Therefore, jejunum is very important in improving growth performance. Whether the jejunal microbiota affects chicken growth performance by reducing intestinal inflammation becomes an interesting question.

To tackle this question, chickens from the same group with different growth performance were used in the present study to compare inflammation levels and jejunal microbial communities. The correlation between inflammation levels and jejunal microbiota was analyzed using Spearman correlation analysis. To verify whether chicken jejunal microbiota potentiated growth performance by reducing intestinal inflammation, transferring fecal microbiota from adult chickens with high body weight into 1-day-old chicks was performed.

## Results

### Different growth performance of high and low body weight chickens

**Fig. 1:**
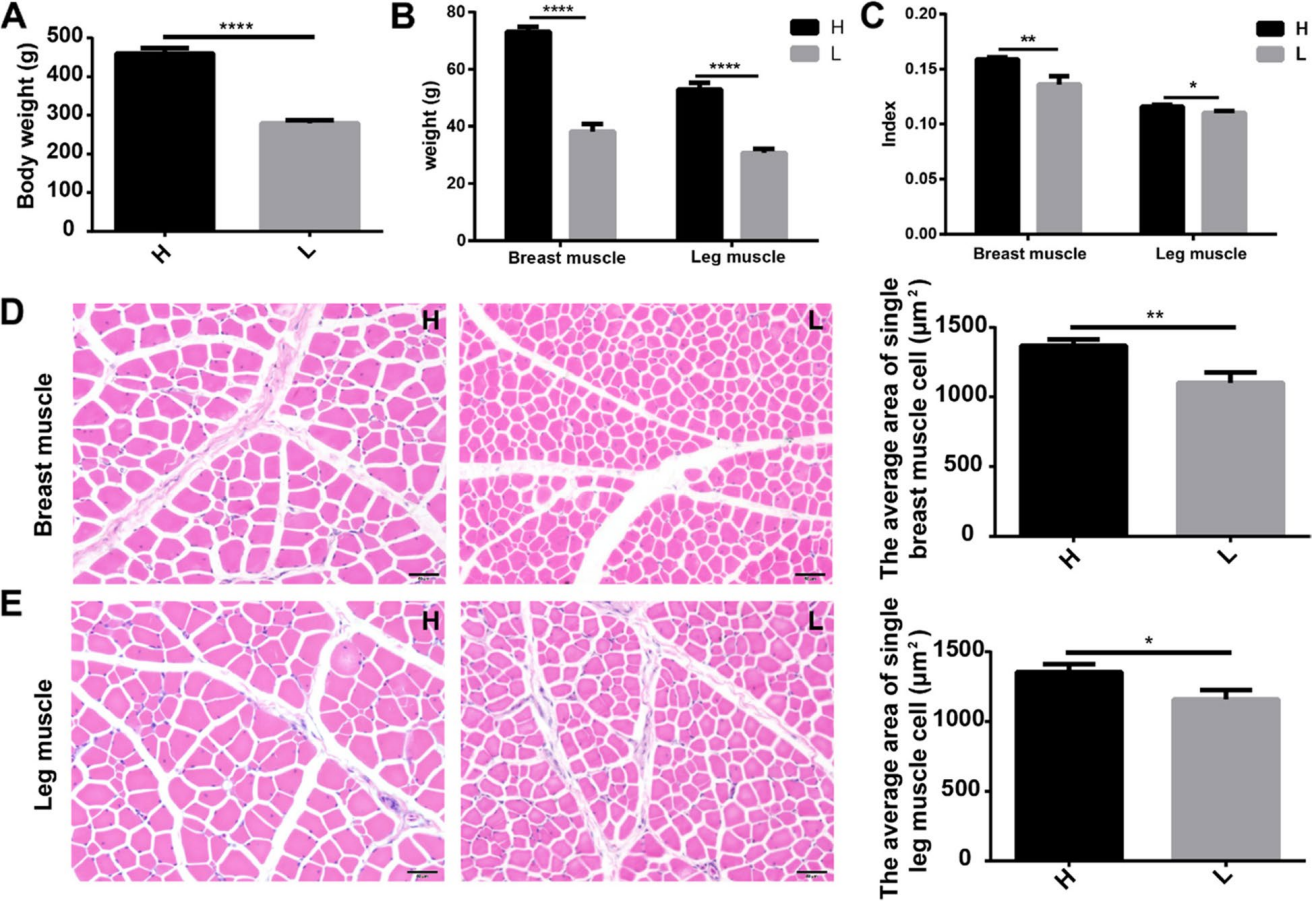
Differential growth performance of high and low body weight chickens. **A** Body weight of high and low groups. **B** Breast and leg muscle weight. **C** Breast and leg muscle indices. **D**, **E** H & E staining of paraffin sections of breast muscles and leg muscles and the comparison of single cell’s cross-sectional area. Scale bars = 50 μm. Data are shown as mean ± SEM. *P < 0.05, **P < 0.01, and ****P < 0.0001. H, high body weight group; L, low body weight group

Seven-week-old chickens with significantly different body weights (H vs L, 460.82 ± 13.22 g vs 278.92 ± 8.24 g, *P* < 0.0001) were selected for subsequent analysis (Fig. 1A). For high (H) vs low (L) body weight chickens, both the mean breast muscle weight (73.09 ± 1.72 g vs 38.17 ± 2.68 g) and leg muscle weight (52.87 ± 2.39 g vs 30.74 ± 1.44 g) were significantly (*P* < 0.0001) different (Fig. 1B). Similarly, both the breast muscle index (0.16 ± 0.002 vs 0.14 ± 0.007, *P* < 0.01) and the leg muscle index (0.12 ± 0.002 vs 0.11 ± 0.002, *P* < 0.05) were significantly larger in high body weight chickens (Fig. 1C). The results of hematoxylin and eosin (H & E) staining indicated that both the average cross-sectional area of single breast muscle cells (1367.68 ± 45.59 μm^2^ vs 1102.22 ± 73.80 μm^2^, *P* < 0.01) (Fig. 1D) and single leg muscle cells (1352.68 ± 57.63 μm^2^ vs 1159.76 ± 67.35 μm^2^, *P* < 0.05) (Fig. 1E) were much larger in high body weight chickens compared with low body weight chickens.

### Differences of jejunal microbiota between high and low body weight chickens

**Fig. 2:**
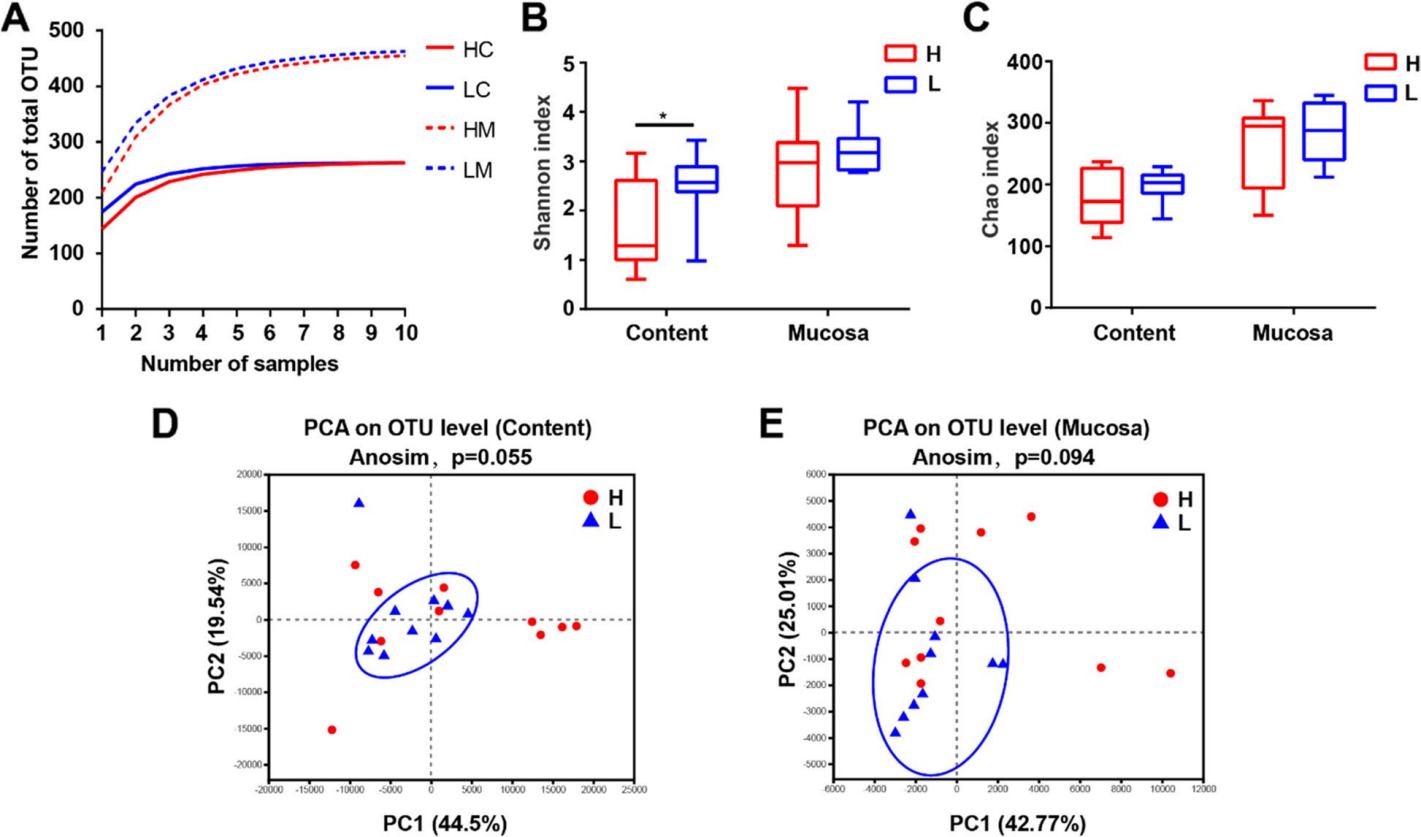
Comparison of microbial *α* diversity and *β* diversity in jejunum between high and low body weight chickens. **A** Pan curve indicates the relation between total number of operational taxonomic unit (OTUs) and the number of samples. **B** Microbial community diversity (measured by Shannon index). **C** Microbial community abundance (measured by Chao index). **D**, **E** Principal component analysis (PCA) plots of Bray–Curtis dissimilarities between the content/mucosa microbiota of high and low body weight groups. HC, the content of high body weight group; LC, the content of low body weight group; HM, mucosa of high body weight group; LM, mucosa of low body weight group

The microbiota of jejunal content and mucosa was analyzed by 16S rRNA gene sequencing. A total of 1,057,457 high-quality reads were obtained from 20 content samples (an average of 52,872 reads per sample), and 995,853 high-quality reads were obtained from 20 mucosal samples (an average of 49,793 reads per sample). The increasing trend of total operational taxonomic units (OTUs) tended to be horizontal as the number of samples increased (Fig. 2A). The microbial diversity in the jejunal content of low body weight chickens (Shannon index ranged from 0.98 to 3.42, median = 2.57) was significantly higher than that in high body weight chickens (Shannon index ranged from 0.60 to 3.16, median = 1.29) (*P* < 0.05) (Fig. 2B). Further, there was no significant difference in total microbial abundance both in jejunal content (H vs L, Chao index ranged from 114.25 to 237.0, median = 172.66 vs 144.0 to 229.25, median = 203.02) and mucosa (H vs L, Chao index ranged from 150.07 to 336.22, median = 294.53 vs 212.25 to 344.53, median = 287.37) (Fig. 2C). The microbiota structure tended to be different, and samples cluster of the low body weight chickens were in a closer distance than that of the high body weight chickens (Fig. 2D, E).

**Fig. 3:**
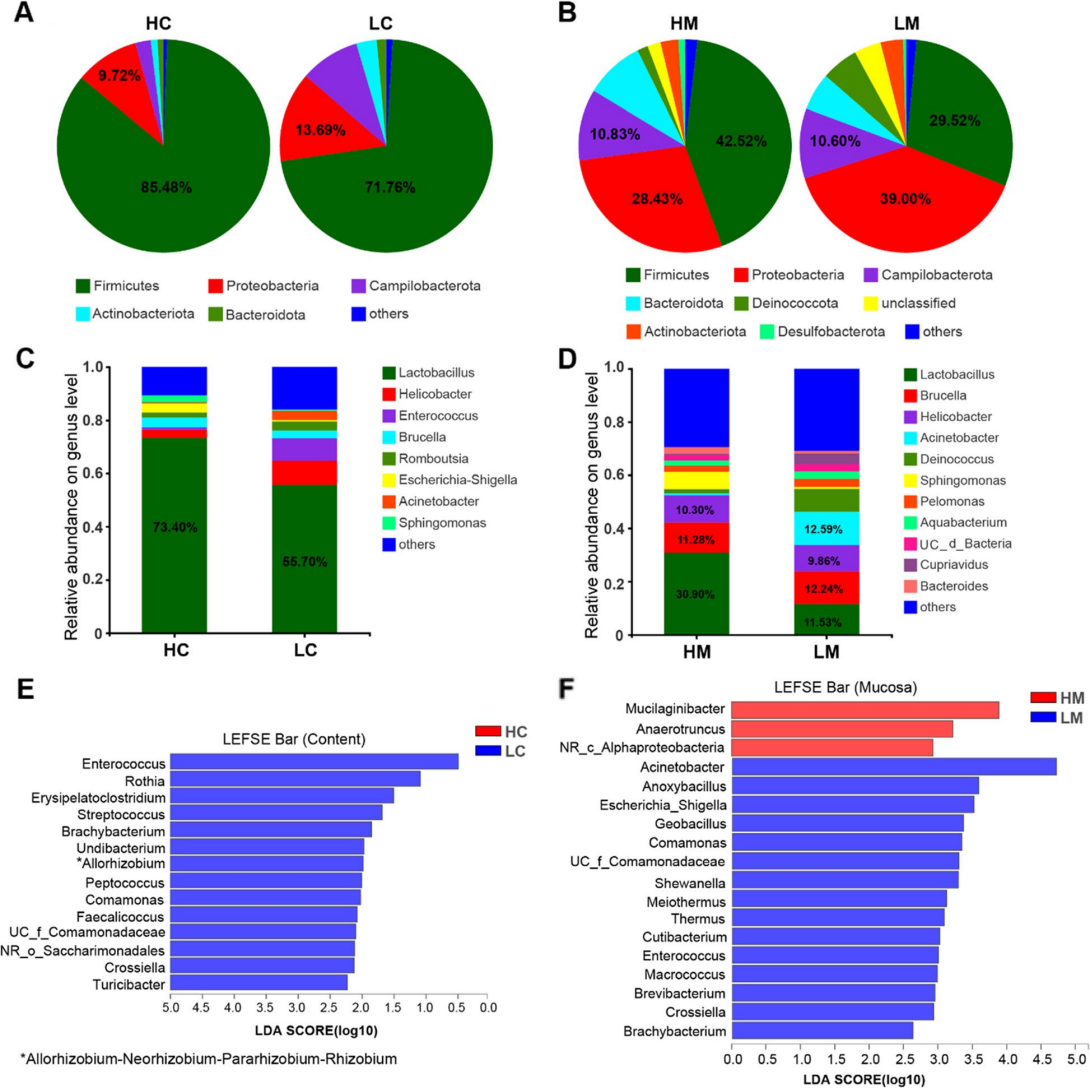
Differences in abundance and microbial composition. **A**, **B** Microbial community composition of jejunum content/mucosa at the phylum level. **C**, **D** Microbial community composition of jejunum content/mucosa at the genus level. **E**, **F** Differentially abundant taxa of content/mucosa microbiota between high and low body weight chickens. LDA score ≥ 2. HC, the content of high body weight group; LC, the content of low body weight group; HM, mucosa of high body weight group; LM, mucosa of low body weight group

At the phylum level, Firmicutes, Proteobacteria, and Campilobacterota were the dominant phyla both in content and mucosa. The relative abundance of Firmicutes, of which most bacteria were Gram-positive, was higher both in the content (H vs L, 85.48 vs 71.76%) and mucosa (H vs L, 42.52 vs 29.52%) of the high body weight chickens, while Proteobacteria (Gram-negative) was more abundant both in the content (H vs L, 9.72 vs 13.69%) and mucosa (H vs L, 28.43 vs 39.00%) of the low body weight chickens. Campilobacterota (Gram-negative) was more abundant in the content of low body weight chickens, while its abundance was similar in the mucosa (Fig. 3A, B). At the genus level, the relative abundance of *Lactobacillus* both in the content (H vs L, 73.40 vs 55.70%) and mucosa (H vs L, 30.90 vs 11.53%) was higher in high body weight chickens, the relative abundance of *Helicobacter* (9.03%) and *Enterobacter* (8.44%) was higher in jejunum content of low body weight chickens, and the relative abundance of *Acinetobacter* (12.59%) and *Deinococcus* (8.61%) was higher in jejunum mucosa of low body weight chickens (Fig. 3C, D). The linear discriminant analysis effect size (LEfSe) analysis showed that the relative abundance of the Gram-negative bacteria, including *Allorhizobium-Neorhizobium-Pararhizobium-Rhizobium*, *Undibacterium*, and *Comamonas*, as well as some possibly pathogenic Gram-positive bacteria such as *Enterococcus* and *Streptococcus*, were significantly higher in jejunal content of low body weight chickens (Fig. 3E). In jejunal mucosa, the Gram-negative bacteria including *Acinetobacter*, *Escherichia Shigella*, *Comamonas*, *Meiothermus*, and *Thermus* were also more abundant in low body weight chickens (Fig. 3F). Moreover, function prediction analysis indicated significant differences in immune-related pathways. For instance, a significantly higher expression of nucleotide-binding oligomerization domain (NOD)-like receptor signaling pathway (*P* < 0.01), bacterial invasion of epithelial cells (*P* < 0.01), and antigen processing and presentation (*P* < 0.05) was found both in the jejunal content and mucosa of low body weight chickens compared with high body weight chickens (Fig. S1).

### Lipopolysaccharide (LPS)-elicited jejunal inflammation and TLR4/MyD88/NF-κB expression in jejunum

**Fig. 4:**
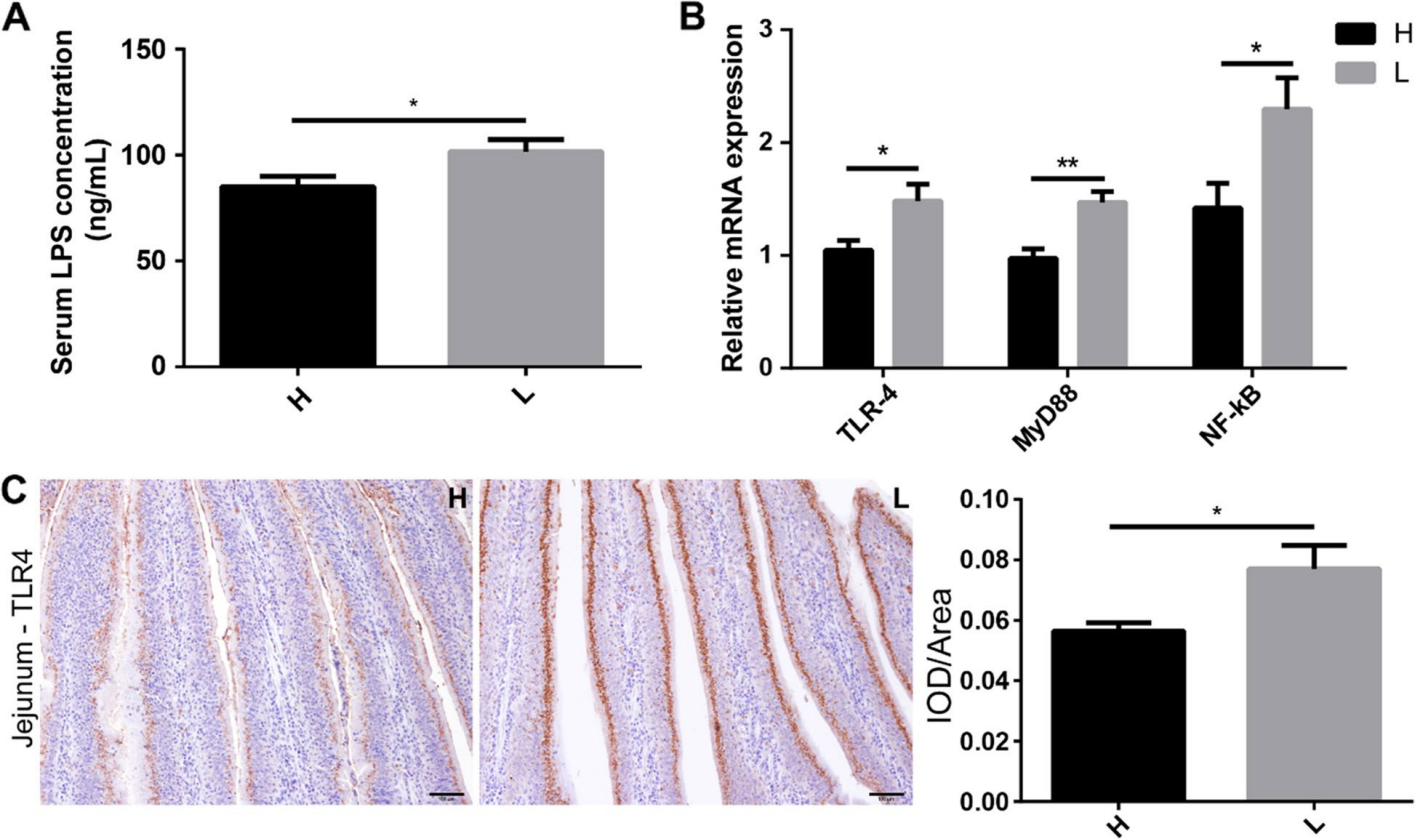
LPS-mediated activation of an inflammatory pathway. **A** Comparison of serum LPS concentrations between high and low body weight chickens. **B** The relative mRNA expression of TLR4, MyD88, and NF-κB in the inflammatory pathway. **C** The protein distribution and expression level of TLR4 in the jejunum (IHC). Data are shown as mean ± SEM. *P < 0.05 and **P < 0.01. IOD, integrated optical density; Scale bars = 100 μm

**Fig. 5:**
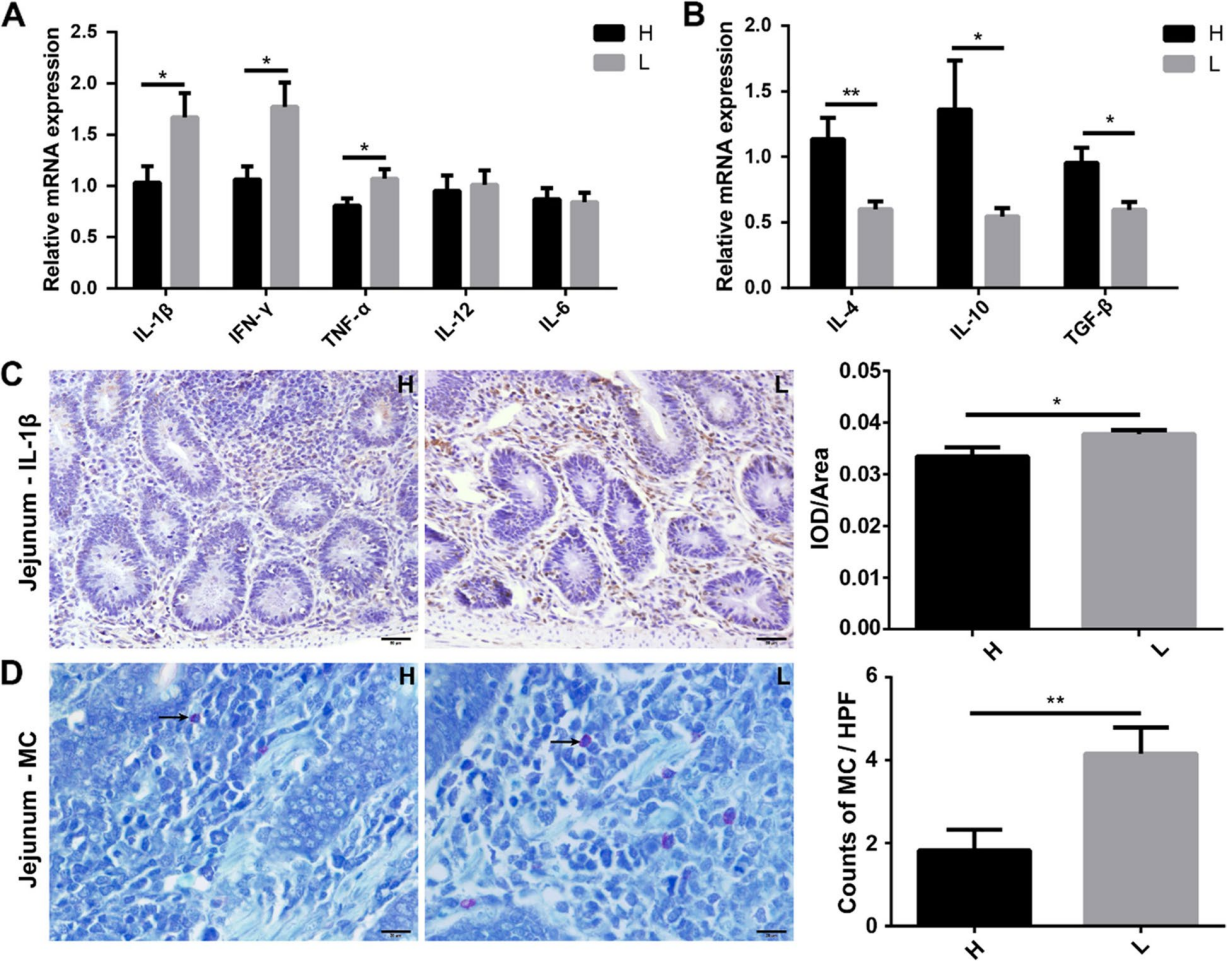
Differential pro-/anti-inflammatory profile. **A** The relative mRNA expression of pro-inflammatory cytokines in the jejunum. **B** The relative mRNA expression of anti-inflammatory cytokines in the jejunum. **C** The protein distribution and expression levels of IL-1β in the jejunum (IHC). Scale bars = 50 μm. **D** Comparison of toluidine blue-stained mast cells in the jejunum. Scale bars = 20μm. Data are shown as mean ± SEM. *P < 0.05, **P < 0.01. IOD, integrated optical density; H, high body weight group; L, low body weight group; MC, mast cell; HPF, high power field

The concentration of serum lipopolysaccharide (LPS) was determined by enzyme-linked immunosorbent assay (ELISA), and inflammatory factors were quantified by quantitative real-time polymerase chain reaction (q-PCR). The results indicated that serum LPS concentration was significantly (*P* < 0.05) higher in low body weight chickens (101.58 ± 5.78 ng/mL) than that in high body weight chickens (85.12 ± 4.79 ng/mL) (Fig. 4A). The relative mRNA expression of Toll-like receptor 4 (TLR4) (H vs L, 1.04 ± 0.09 vs 1.48 ± 0.150, *P* < 0.05), myeloid differentiation factor 88 (MyD88) (H vs L, 0.97 ± 0.08 vs 1.47 ± 0.09, *P* < 0.01), and nuclear factor-kappa B (NF-κB) (H vs L, 1.42 ± 0.22 vs 2.29 ± 0.28, *P* < 0.05) was significantly higher in low body weight chickens compared with high body weight chickens (Fig. 4B). Immunohistochemistry (IHC) results indicated that the protein expression (integrated optical density; IOD)/area of TLR4 was 0.077 ± 0.008 in low and 0.056 ± 0.003 in high body weight chickens, which was also significantly (*P* < 0.05) higher in low body weight chickens (Fig. 4C). The relative mRNA expression of pro-inflammatory factors including interleukin-1β (IL-1β) (H vs L, 1.03 ± 0.16 vs 1.67 ± 0.23), interferon-γ (IFN-γ) (H vs L, 1.06 ± 0.13 vs 1.77 ± 0.24), and tumor necrosis factor α (TNF-α) (H vs L, 0.81 ± 0.07 vs 1.07 ± 0.09) was significantly (*P* < 0.05) higher in low body weight chickens (Fig. 5A). In comparison, the relative mRNA expression of anti-inflammatory factors including interleukin-4 (IL-4) (H vs L, 1.14 ± 0.16 vs 0.60 ± 0.06, *P* < 0.01), interleukin-10 (IL-10) (H vs L, 1.36 ± 0.34 vs 0.55 ± 0.06, *P* < 0.05), and transforming growth factor-β (TGF-β) (H vs L, 0.96 ± 0.11 vs 0.59 ± 0.06, *P* < 0.05) was significantly (*P* < 0.05) higher in high body weight chickens (Fig. 5B). IHC results also indicated significantly (*P* < 0.05) lower expression of IL-1β protein (IOD/area, H vs L, 0.03 ± 0.002 vs 0.04 ± 0.0007) in the jejunum of high body weight chickens (Fig. 5C). The results of toluidine blue staining showed significantly (*P* < 0.01) more mast cells in the jejunum of low body weight chickens (4.16 ± 0.63) than in high body weight chickens (1.82 ± 0.50) (Fig. 5D).

### Intestinal inflammation-induced barrier disruption and apoptosis in the jejunum

**Fig. 6:**
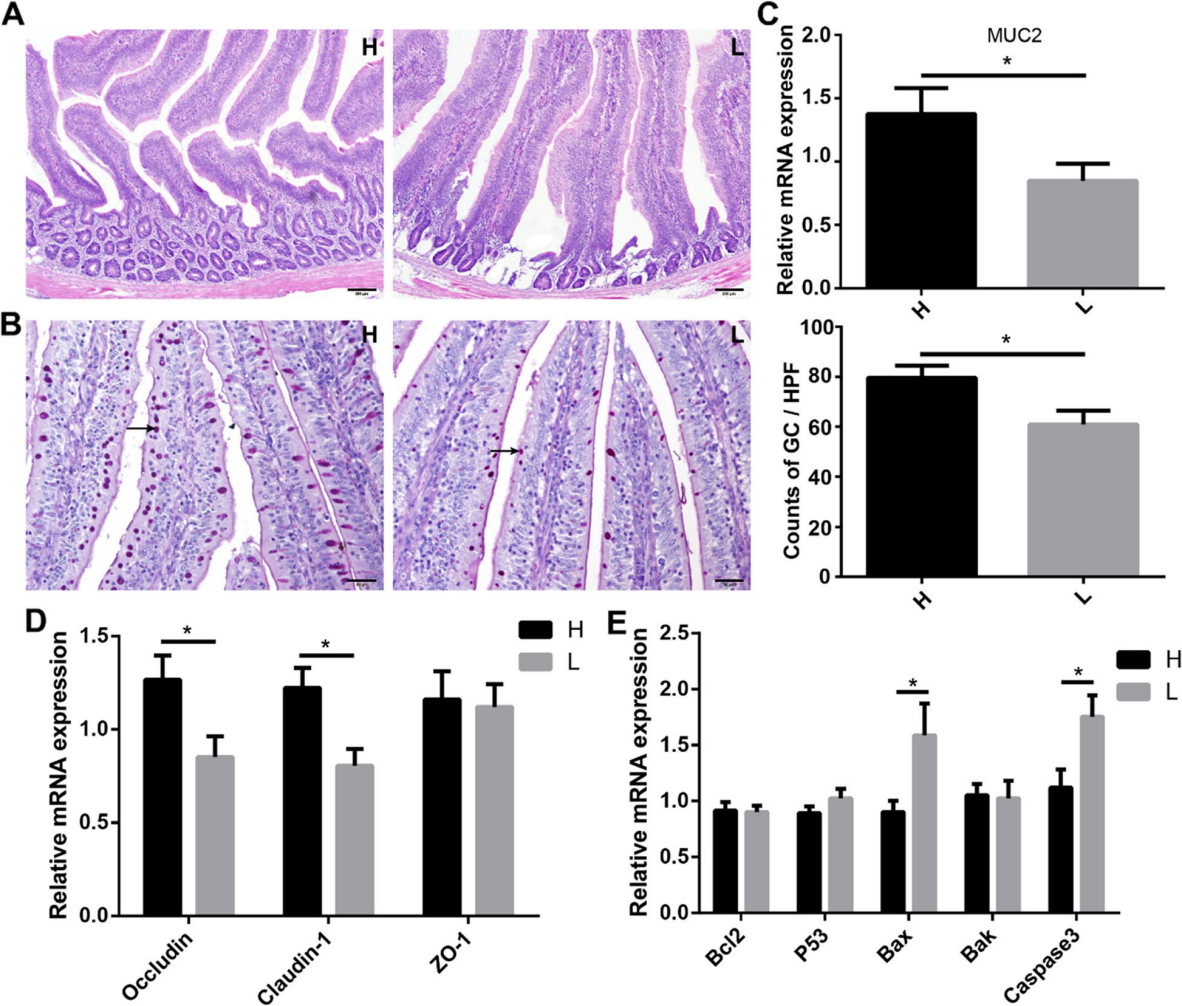
Effect of inflammation and apoptosis on the structure and function of jejunum. **A** H & E staining of the jejunum. Scale bars = 200 μm. **B** Comparison of PAS-stained goblet cells (GC) in the jejunum. Scale bars = 50 μm. **C** The relative mRNA expression of mucin 2 (MUC2) in the jejunum. **D** The relative mRNA expression of tight junction proteins in the jejunum. **E** The relative mRNA expression of apoptosis-related genes. Data are shown as mean ± SEM. *P < 0.05. H, high body weight group; L, low body weight group

The H & E staining results showed that the structure of crypt in the jejunum of low body weight chickens was slightly damaged, and the number of crypts was more in the jejunum of high body weight chickens (Fig. 6A). The Periodic Acid-Schiff (PAS) staining results indicated that the number of goblet cells was significantly (*P* < 0.05) more in the jejunum of high body weight (79.59 ± 4.89) than in low body weight (60.90 ± 5.49) chickens (Fig. 6B). The relative mRNA expression of mucin 2 (MUC2), which was secreted by goblet cells, was significantly (*P* < 0.05) higher in high body weight chickens (1.38 ± 0.20) compared with low body weight chickens (0.85 ± 0.14) (Fig. 6C). The relative mRNA expression of tight junction protein-related genes, i.e., occludin (H vs L, 1.27 ± 0.13 vs 0.85 ± 0.11) and claudin-1 (H vs L, 1.22 ± 0.11 vs 0.81 ± 0.09) was significantly higher in the jejunum of high body weight chickens than in low body weight chickens (*P* < 0.05) (Fig. 6D). There was no significant difference in the relative mRNA expression of zonula occludens 1 (ZO-1) (1.16 ± 0.15 vs 1.12 ± 0.12) in high and low body weight chickens (Fig. 6D). Moreover, the relative mRNA expression of apoptosis-related genes bax (H vs L, 0.90 ± 0.10 vs 1.59 ± 0.23) and caspase 3 (H vs L, 1.12 ± 0.16 vs 1.75 ± 0.19) was significantly (*P* < 0.05) higher in the jejunum of low body weight chickens (Fig. 6E).

### Jejunal pathogens are responsible for intestinal inflammation in chickens

**Fig. 7:**
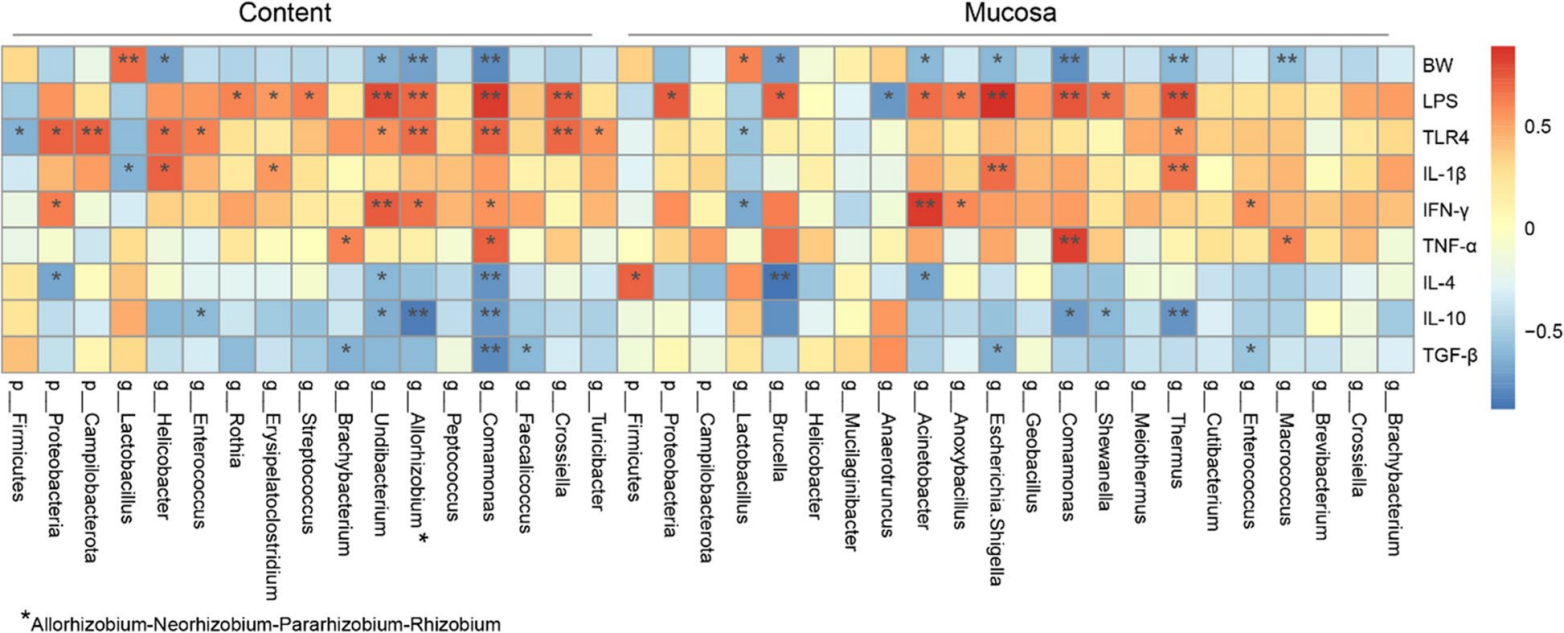
Heatmap of Spearman’s correlations between jejunal microbiota abundance and phenotype/inflammatory factors. The colors range from blue (negative correlation) to red (positive correlation). *P < 0.05 and **P < 0.01

Spearman correlation analysis was used to analyze the correlation between inflammation-related factors, body weight, and differential gut microbiota. The results suggested that the abundance of *Lactobacillus* was significantly (*P* < 0.05) and positively correlated with body weight, while the abundance of *Comamonas*, *Acinetobacter*, *Brucella*, *Escherichia-Shigella*, *Thermus*, *Undibacterium*, and *Allorhizobium-Neorhizobium-Pararhizobium-Rhizobium* was significantly (*P* < 0.05) and negatively correlated with the body weight and the relative mRNA expression of anti-inflammatory factors, i.e., IL-4, IL-10, and TGF-β. Conversely, these bacteria were significantly (*P* < 0.05) and positively correlated with the relative mRNA expression of pro-inflammatory factors, i.e., IL-1β, IFN-γ, and TNF-α (Fig. 7).

### FMT improved chicken growth performance

**Fig. 8:**
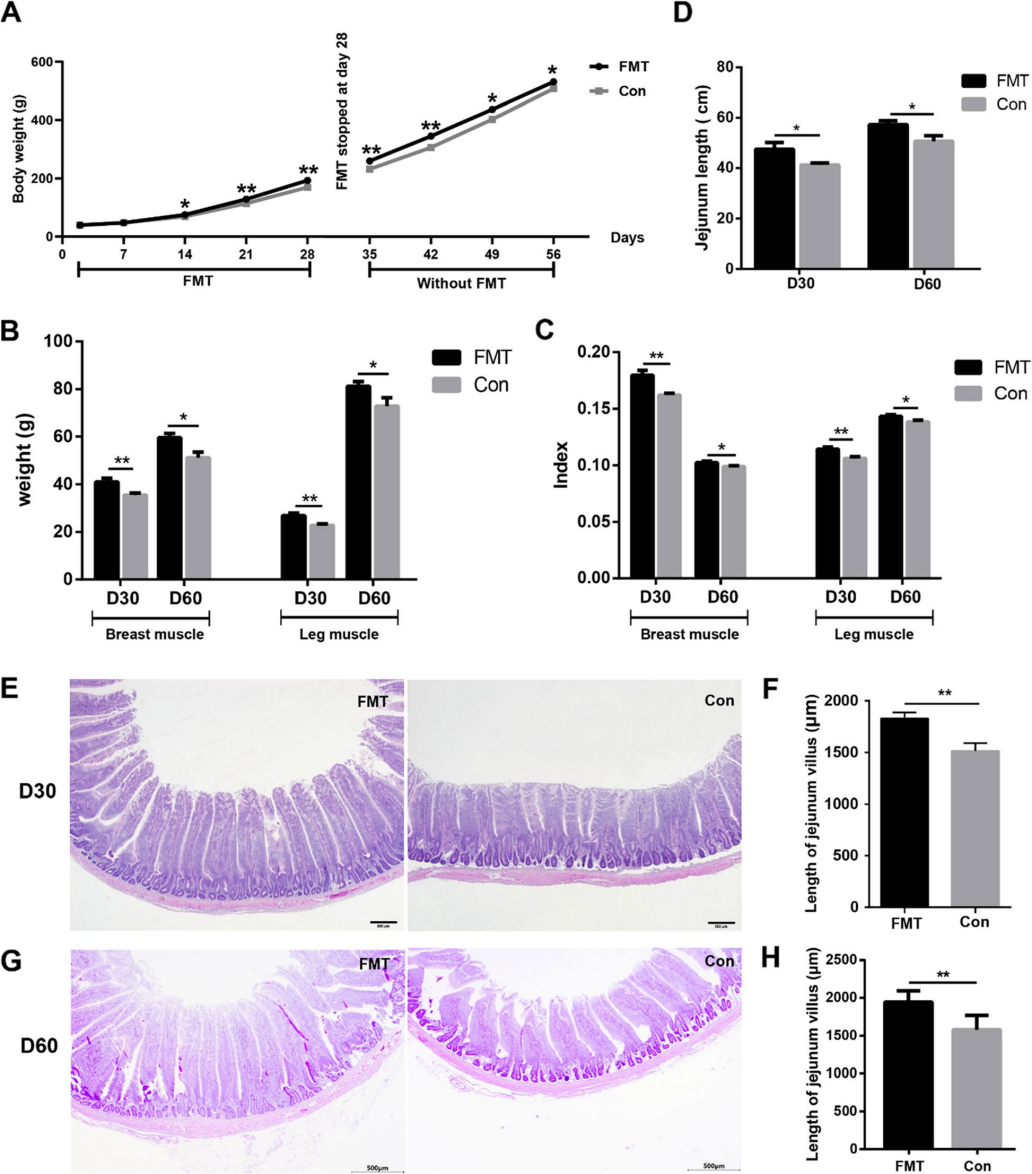
Effect of fecal microbiota transplantation (FMT) on chicken growth and development. **A** FMT promoted the weight gain of chickens. **B** Breast muscle and leg muscle weight on the 30th and 60th day. **C** Breast muscle and leg muscle indices on the 30th and 60th day. **D** Comparison of the jejunum length on the 30th and 60th day. **E** The difference of jejunum epithelial morphology of FMT and control group chickens on the 30th day. Scale bars = 500 μm. **F** The difference in jejunum villus height between FMT and control group chickens on the 30th day. **G** The difference of jejunum epithelial morphology of FMT and control group chickens on the 60th day. Scale bars = 500 μm. **H** The difference in jejunum villus height between FMT and control group chickens on the 60th day. Data are shown as mean ± SEM. *P < 0.05 and **P < 0.01. FMT, fecal microbiota transplantation group; Con, control group

FMT was performed to investigate the effects of gut microbiota on growth performance and inflammation factors. The results indicated that the body weight on the 14th day (FMT vs Con, 75.48 ± 1.45 g vs 69.15 ± 2.18 g, *P* < 0.05), 21st day (FMT vs Con, 128.83 ± 3.32 g vs 114.29 ± 3.41 g, *P* < 0.01), and 28th day (FMT vs Con, 193.23 ± 4.19 g vs 169.67 ± 5.37 g, *P* < 0.01) was significantly higher in the FMT group compared with the control group (Con) (Fig. 8A). We stopped FMT treatment on the 30th day, and twenty chickens were sacrificed (ten chickens from each group), but the other chickens were reared continuously without FMT treatment and were sacrificed on the 60th day. Interestingly, the mean body weight on the 35th day (FMT vs Con, 259.12 ± 4.82 g vs 231.03 ± 8.34 g, *P* < 0.01), 42th day (FMT vs Con, 344.60 ± 7.32 g vs 305.75 ± 12.12 g, *P* < 0.01), 49th day (FMT vs Con, 436.24 ± 7.24 g vs 402.20 ± 10.68 g, *P* < 0.05), and 56th day (FMT vs Con, 530.62 ± 8.73 g vs 496.80 ± 11.82 g, *P* < 0.05) was still significantly higher in FMT group (Fig. 8A). The breast muscle weight both on the 30th day (FMT vs Con, 41.05 ± 1.55 g vs 35.46 ± 0.91 g, *P* < 0.01) and 60th day (FMT vs Con, 59.52 ± 1.89 g vs 51.19 ± 2.38 g, *P* < 0.05), and the leg muscle weight both on the 30th day (FMT vs Con, 26.80 ± 1.05 g vs 22.83 ± 0.59 g, *P* < 0.01) and 60th day (FMT vs Con, 81.25 ± 1.85 g vs 72.85 ± 3.53 g, *P* < 0.05) were significantly larger in the FMT group as well (Fig. 8B). Besides, the breast muscle indices both on the 30th day (FMT vs Con, 0.18 ± 0.004 vs 0.16 ± 0.002, *P* < 0.01) and 60th day (FMT vs Con, 0.102 ± 0.001 vs 0.099 ± 0.001, *P* < 0.05), and the leg muscle indices both on the 30th day (FMT vs Con, 0.114 ± 0.002 vs 0.106 ± 0.001, *P* < 0.01) and 60th day (FMT vs Con, 0.143 ± 0.002 vs 0.138 ± 0.001, *P* < 0.05) were significantly larger in FMT group (Fig. 8C). The jejunum length both on the 30th day (FMT vs Con, 47.50 ± 2.69 cm vs 41.20 ± 0.85 cm) and 60th day (FMT vs Con, 57.33 ± 1.52 cm vs 50.67 ± 2.23 cm) were significantly longer in FMT group (Fig. 8D). Further, the length of jejunum villus both on the 30th day (FMT vs Con, 1829 ± 58.62 μm vs 1516 ± 77.03 μm, *P* < 0.01) (Fig. 8E, F) and on the 60th day (FMT vs Con, 1943 ± 61.09 μm vs 1580 ± 77.54 μm, *P* < 0.01) (Fig. 8G, H) were significantly longer in the FMT group.

### FMT reshaped jejunal microbiota and mitigated inflammatory response

**Fig. 9:**
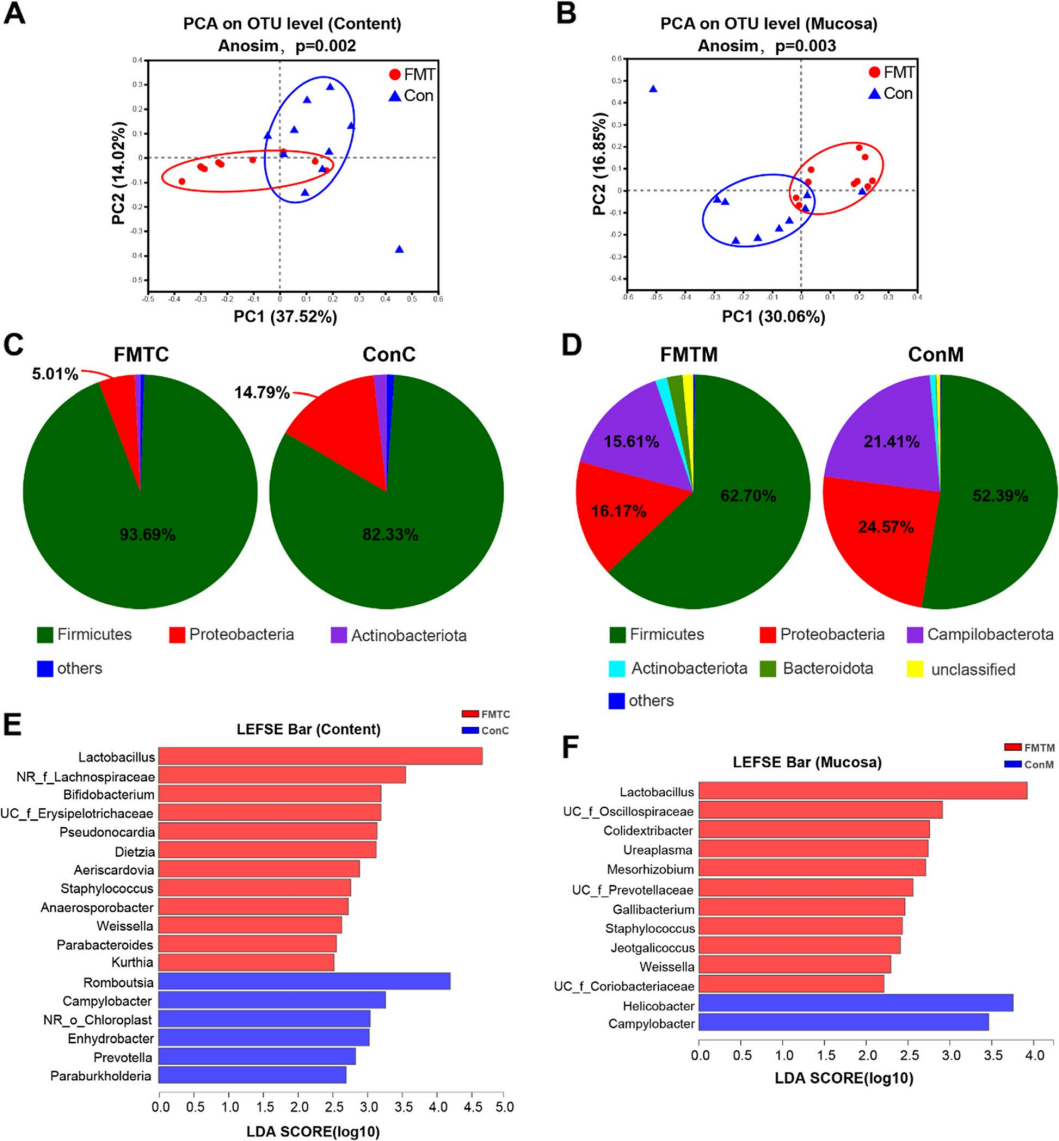
Effect of fecal microbiota transplantation (FMT) on jejunum microbiota. **A**, **B** Principal component analysis (PCA) plots of Bray–Curtis dissimilarities between the content/mucosa microbiota of the FMT and control groups. **C**, **D** Microbial community composition of jejunum content/mucosa at the phylum level. **E**, **F** Differentially abundant taxa of content/mucosa microbiota between FMT and control groups. LDA score ≥ 2. FMTC, the content of the fecal microbiota transplantation group; ConC, the content of the control group; FMTM, mucosa of the fecal microbiota transplantation group; ConM, mucosa of the control group

**Fig. 10:**
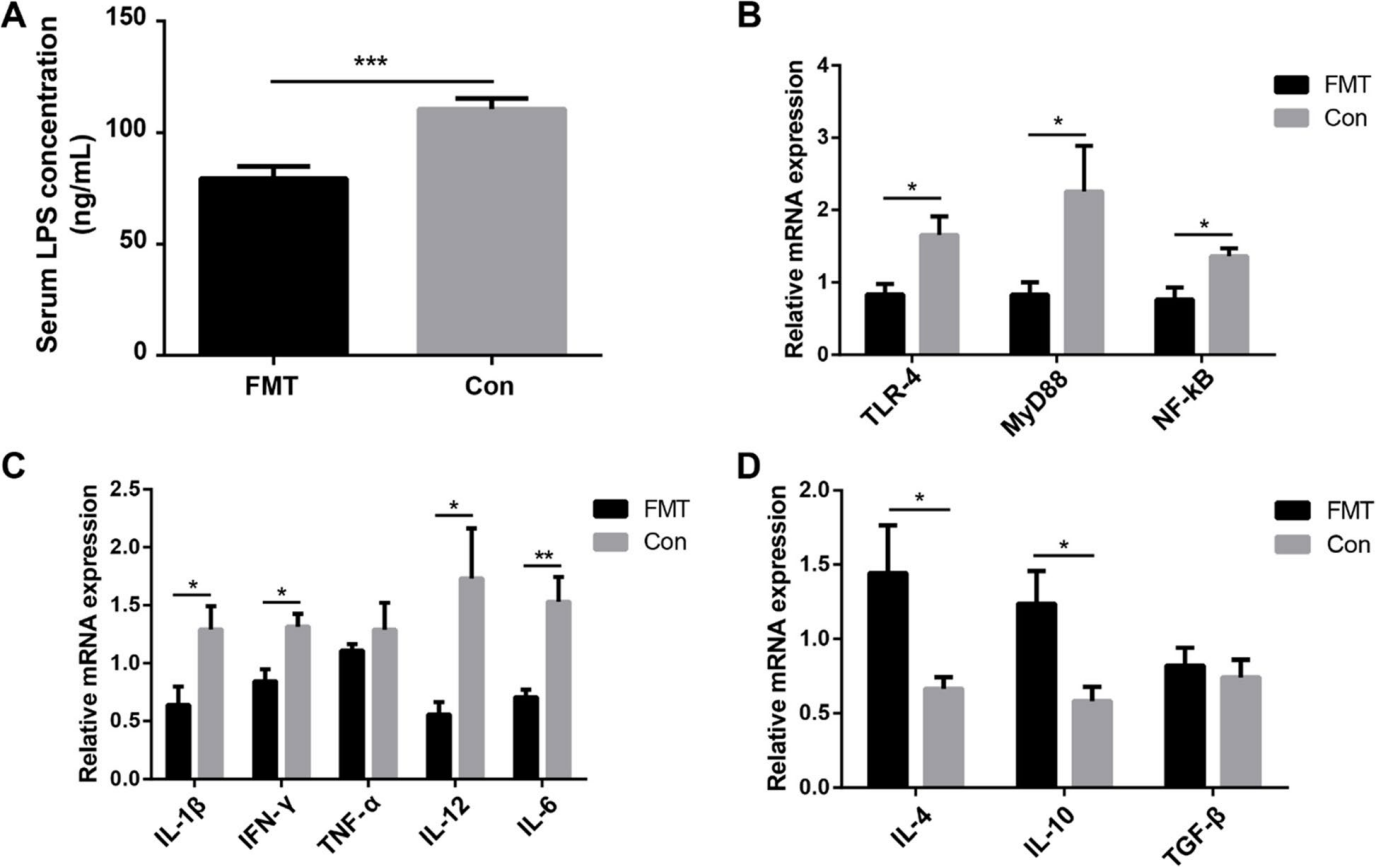
Effect of fecal microbiota transplantation (FMT) on inflammatory pathways. **A** Serum LPS concentration in the FMT and control groups. **B** The relative mRNA expression of TLR4 MyD88 and NF-κB in the jejunum. **C** The relative mRNA expression of pro-inflammatory cytokines in the jejunum. **D** The relative mRNA expression of anti-inflammatory cytokines in the jejunum. Data are shown as mean ± SEM. *P < 0.05, **P < 0.01, and ***P < 0.001

The microbiota of jejunal content and mucosa in the FMT experiment was also analyzed by 16S rRNA gene sequencing. The principal component analysis (PCA) results showed a significant difference in microbiota structure both in jejunal content and mucosa between the FMT and the control groups (Fig. 9A, B). At the phylum level, the relative abundance of Firmicutes was higher both in the content (FMT vs Con, 93.69 vs 82.33%) and mucosa (FMT vs Con, 62.70 vs 52.39%) of the FMT group chickens. Proteobacteria was more abundant both in the content (FMT vs Con, 5.01 vs 14.79%) and mucosa (FMT vs Con, 16.17 vs 24.57%) of the control group chickens. Similarly, the relative abundance of Campilobacterota was lower in the jejunal mucosa (FMT vs Con, 15.61 vs 21.41%) of the FMT group chickens (Fig. 9C, D). Linear discriminant analysis effect size (LEfSe) analysis exhibited that the relative abundance of *Lactobacillus* and *Bifidobacterium* was significantly higher both in the content and mucosa of the FMT group chickens. In comparison, the relative abundance of *Campylobacter* was significantly higher both in the content and mucosa of the control group chickens (Fig. 9E, F). ELISA results indicated that the serum LPS concentration (FMT vs Con, 79.39 ± 5.41 ng/mL vs 110.55 ± 4.78 ng/mL, *P* < 0.001) was significantly lower in the FMT group (Fig. 10A). Correspondingly, the relative mRNA expression of TLR4 (FMT vs Con, 0.83 ± 0.15 vs 1.66 ± 0.25), MyD88 (FMT vs Con, 0.83 ± 0.17 vs 2.25 ± 0.63), and NF-κB (FMT vs Con, 0.76 ± 0.17 vs 1.36 ± 0.11) was significantly (*P* < 0.05) lower in the jejunum of the FMT group (Fig. 10B). Likewise, the relative mRNA expression of pro-inflammatory cytokines, i.e., IL-1β (FMT vs Con, 0.64 ± 0.16 vs 1.29 ± 0.20, *P* < 0.05), IFN-γ (FMT vs Con, 0.85 ± 0.10 vs 1.32 ± 0.11, *P* < 0.05), IL-12 (FMT vs Con, 0.56 ± 0.10 vs 1.73 ± 0.43, *P* < 0.05), and IL-6 (FMT vs Con, 0.71 ± 0.06 vs 1.53 ± 0.21, *P* < 0.01) was significantly lower in the jejunum of the FMT group (Fig. 10C). On the other hand, the relative mRNA expression of anti-inflammatory cytokines, i.e., IL-4 (FMT vs Con, 1.44 ± 0.32 vs 0.67 ± 0.07, *P* < 0.05), and IL-10 (FMT vs Con, 1.24 ± 0.22 vs 0.59 ± 0.09, *P* < 0.05) was significantly higher in the jejunum of the FMT group (Fig. 10D).

## Discussion

Intestinal inflammation is closely associated with chicken growth performance [27]. In the present study, the inflammation response level was significantly higher in low body weight chickens, suggesting higher intestinal inflammation level leads to lower growth performance. It is hypothesized that early colonization of beneficial microbiota in the gut could increase growth performance, yet early colonization of harmful microbiota could cause intestinal inflammation, destroy the intestinal structure, affect nutrient uptake, and finally resulted in compromised growth performance. An abrupt compositional shift in chicken gut microbiota directs to dysbiosis, which increases the pathogenic abundance, especially Gram-negative bacteria [28, 29]. For instance, Proteobacteria belongs to the phylum of Gram-negative bacteria and also abundantly present in the chicken intestine [30]. Some species of these genera (*Comamonas*, *Acinetobacter*, *Brucella, Shigella*, and *Escherichia* coli (*E. coli*), etc.), which belong to Proteobacteria, could be pathogenic and cause apparent signs of gut diseases. Thus, the increased relative abundance of Proteobacteria leads to disease development and reduces chicken growth performance [31, 32]. In our results, the abundance of Proteobacteria both in jejunal content and mucosa was negatively associated with the body weight gain. *Comamonas*, a Gram-negative pathogenic bacterium, can cause degradation of steroid hormones [33, 34], is predominately associated with bacteremia [35], and can occasionally cause low virulence diseases in human and animals [36]. A recent study demonstrated that a higher abundance of *Acinetobacter* markedly increased systemic inflammation via inducing IFN-γ and IL-6 production [37] and resulted in poor efficiency in broilers [38]. Another study recognized that the pathogenic *Helicobacter pylori*, *E. coli*, and *Brucella spp*. remarkably decreased the broiler’s growth performance [39]. Researchers have also observed a higher abundance of *Escherichia-Shigella* in intestinal inflammation, and its dissemination imparted toxic effects on chicken growth [40]. Although literature availability for *Thermus*-induced inflammation is limited, a human study described its negative correlation with serum IL-10, suggesting its role in inflammation [41]. Additionally, Sun et al. [42] detected *Undibacterium* as an abundant (29.67%) intestinal bacterium, and it was also found during the peak of disease [43]. *Allorhizobium-Neorhizobium-Pararhizobium-Rhizobium* was also observed as an endemic in the duck cecum and is speculated to cause disease [44]. In the present study, the above Gram-negative bacteria were more abundant both in the content and mucosa of low body weight chickens, resulting in intestinal inflammation and reduced growth performance. On the other hand, gut microbiota also helps protect the broilers via attenuating intestinal inflammation [45] and reducing the colonization of harmful bacteria [46]. Firmicutes are principally documented as the largest gut microbial component [8, 47], the predominant species among 500 bacterial species in the chicken intestine [48], and significantly and positively associated with improved chicken weight gain [49–51]. *Lactobacilli* and *Bifidobacteria* are purported beneficial for gut physiology and body weight gain and effective in gut inflammation [52, 53]. Drissi et al. [54] and co-workers reported several *Lactobacillus* species as a masterpiece affecting weight gain both in human and animals. The increased abundance of *Lactobacilli* and *Bifidobacteria* is related to higher chicken’s body weight and vice versa [55]. Another report described that *Lactobacilli* positively improved the intestinal mucosa, strengthened gut barrier, competed with *E. coli* for colonization, and modulated inflammatory response [56]. Our results were in line with the above findings, as we found that high body weight chickens harbored abundant Firmicutes and *Lactobacilli* both in jejunal content and mucosa.

It has been established that Gram-negative bacteria contribute to the release of LPS, which induces the expression of inflammatory cytokines via TLR4-mediated MyD88 and NF-κB pathways [57, 58]. In the present study, the serum LPS concentration was significantly higher in low body weight chickens, suggesting more Gram-negative bacteria released more LPS, which triggers the production of inflammatory cytokines via TLR4/MyD88/NF-κB pathways. It has been reported that the expression of TLR4, MyD88, and NF-κB in the chicken intestinal epithelium during intestinal inflammation was remarkably upregulated [59, 60], which was consistent with our results. Subsequently, activated NF-κB translocates into the nucleus and induces secretion of multiple pro-inflammatory cytokines, i.e., IL-1β, IL-6, and TNF-α [61]. Overwhelming production of pro-inflammatory cytokines is the indicator of inflammation, and an elevated level of anti-inflammatory cytokines is the extent of eliminating inflammation via effective host immune response during chicken gut inflammation [62–64]. Consistent with these findings, in our study, a significantly elevated relative mRNA expression of pro-inflammatory cytokines (IL-1β, IFN-γ, TNF-α, IL-12, and IL-6) in the low body weight chicken’s jejunum indicated the increased intestinal inflammation. Conversely, an evidently increased relative mRNA expression of the anti-inflammatory cytokines (IL-4, IL-10, and TGF- β) in the high body weight chicken’s jejunum suggested the decreased intestinal inflammation. Mast cells activation involves in the pathogenesis of gut inflammation [65], and its count is remarkably raised in the chicken during intestinal inflammation [66]. A higher number of mast cells during Gram-negative infection in the chicken intestine [67] is consistent with our findings. Besides, mast cells can be activated through TLRs/NF-κB pathway and produce pro-inflammatory cytokines during intestinal inflammation [68], suggesting an essential role of mast cells in governing the intense inflammatory response in low body weight chickens.

Increased inflammation destroyed the jejunal structure in broilers and impaired the integrity of the tight junctions [40, 69]. Thus, preventing pathogens’ translocation from chicken jejunum into the systemic circulation is compromised [70]. In the present study, H & E and PAS staining and qPCR results indicated the disrupted jejunal structure in low body weight chickens. Previously, Chen et al. [71] reported that impaired tight junctions’ proteins resulted in gut barrier dysfunctions, and LPS is the leading cause of this impairment. Recently, Yu et al. [72] described that LPS remarkably induced loss of goblet cells, thus decreased the mucin 2 production and compromised the integrity of the gut mucus blanket. Consistent with these findings, we also found comparatively lower mRNA expression of mucin 2 and less number of goblet cells in the low body weight chicken’s jejunum. These results suggested decreased goblet cells and reduced production of intestinal mucin 2 in low body weight chicken’s jejunum. Consistently, our results demonstrated that relative mRNA expression of occludin and claudin-1 in low body weight chicken’s jejunum was significantly decreased compared with high body weight chickens. Caspase-3 plays an executive role in apoptosis, and bax and bcl-2 are the chief regulators of apoptosis. Bax is characterized as an apoptotic promoter, while bcl-2 is an apoptotic suppresser [60]. Likewise, our results found significantly increased relative mRNA expression of pro-apoptotic protein bax and caspace-3 in low body weight chicken’s jejunum, signifying an inevitable occurrence of apoptosis and suggesting a deleterious influence of LPS on intestinal health. Our findings are also in agreement with Yang et al. [69], who demonstrated apoptosis status with elevated mRNA expression of a pro-apoptotic gene bax in broiler jejunum during intestinal inflammation.

Intestinal inflammation can be mitigated by reshaping gut microbiota [73], which enhances the intestinal health of poultry [74]. Presently, dietary interventions and probiotic supplementation are regarded as essential to alter the gut microbiota, and FMT has got considerable attention to reshape the host intestinal microbiota via assessing the microbe-host signal profile for improving human and chicken health [19, 75, 76]. *Lactobacillus* and *Bifidobacterium* are the potential probiotics that prominently influence the chicken microbial abundance [46], and Yang et al. [69] demonstrated that supplementation of *Lactobacillus spp.* could considerably stabilize the microbiota community by manipulating the chicken gut microenvironment and substantially alleviate intestinal inflammation. A recent study in the humans indicated that FMT could remarkably increase the abundance of *Bifidobacterium* and *Lactobacillus* and reduce pathogen colonization in the infant’s gut [77]. Li et al. [78] demonstrated the downregulation of NF-κB and pro-inflammatory cytokines using a probiotic (*Lactobacillus*) supplementation in broilers during intestinal inflammation. In the present study, FMT significantly increased the relative abundance of *Lactobacillus* and *Bifidobacterium* both in the jejunal content and mucosa, decreased the pro-inflammatory cytokine (IL-1β, IFN-γ, IL-12, and IL-6) levels, and enhanced the anti-inflammatory cytokine (IL-4 and IL-10) levels, indicating FMT mitigated intestinal inflammation by reshaping unbalanced gut microbiota.

## Conclusions

Taken together, early colonization of harmful bacteria especially Gram-negative bacteria (*Comamonas*, *Acinetobacter*, *Brucella*, *Escherichia-Shigella*, *Thermus*, *Undibacterium*, and *Allorhizobium-Neorhizobium-Pararhizobium-Rhizobium*) in the chicken jejunum releases more LPS, which induces the expression of inflammatory cytokines via TLR4-mediated MyD88 and NF-κB pathways resulting in intestinal inflammation, while early colonization of beneficial bacteria especially Gram-positive bacteria (*Lactobacillus* and *Bifidobacterium*) could enhance the anti-inflammatory cytokine levels. FMT could reshape jejunal microbiota, mitigate intestinal inflammation, and improve chicken growth performance. FMT could be a potential strategy to improve animal growth performance.

## Methods

### Animals

The Institutional Animal Care and Use Committee of Huazhong Agricultural University (HZAUCH-2018-008), Wuhan, China) approved all the animal procedures, and all methods were performed in accordance with the relevant guidelines and regulations.

Newly hatched chickens (Turpan cockfighting × White Leghorn chickens both for meat and eggs) were reared under similar husbandry conditions in the metal cages at a density of 10 chickens per cage in the poultry farm of Huazhong Agricultural University. The chickens were fed a corn-soybean diet in pellet form with no medication or vaccination. The birds had ad libitum access to water and feed. At the age of 7 weeks, all 200 chickens were weighed, then a total of twenty chickens with the highest (H) body weight (*n*=10, five males and five females) or the lowest (L) body weight (*n*=10, five males and five females) were selected for the next study.

For FMT experiment, two adult female chickens in the same batch were selected as fecal donors. In the morning, once the donor chickens defecated, the white part of the excreta was removed immediately because it mainly comprises uric acid. Feces (8 g) were collected daily in the sterile tube (50 mL) and mixed with 0.75% saline in 1:6 ratios (6 mL of 0.75% saline for each gram of feces). Keeping the mixture on ice until precipitates were fully settled down, the supernatant was collected and filtered with the sterile gauze to get fecal suspension. A total of 60 1-day-old chicks with the same genetic background were selected as recipients and randomly divided into the FMT group and control group. Birds in the FMT group were orally administrated with 1-mL fecal microbiota suspension, while 0.75% saline was used as a substitute in the control group for 30 days. At 30th day, twenty chickens were sacrificed (ten chickens from each group), and samples were collected. Other chickens were reared continuously but without FMT treatment. At 60th day, the forty chickens (twenty chickens from each group) were sacrificed and samples were collected.

### Sample collection

After fasting for 12 h, the chickens were sacrificed, and the blood, breast muscle, leg muscle, and jejunum were harvested. For gut microbiota analysis, the gastrointestinal tract was rapidly removed and the jejunal segment (about 13 to 15 cm per bird) was excised. The jejunal content (1 to 1.5 g per bird) was collected into two sterilized centrifuge tubes (1.5 mL). Then, the excised jejunal segment was cut open and flushed gently with the sterilized normal saline (0.75%), and the mucosa (0.6 to 0.8 g per bird) was collected into two sterilized centrifuge tubes (1.5 mL) by scraping the jejunal segment with the sterilized tweezer. The contents and mucosae of jejunum were snap-frozen in liquid nitrogen, then stored at −80°C for sequencing. For histo-morphological analysis, freshly harvested the breast muscles, leg muscles, and jejunum tissues were fixed in 4% paraformaldehyde solution. For gene expression analysis, the parts of freshly harvested jejunum tissues were snap-frozen in liquid nitrogen and then stored at −80°C. For analysis of LPS concentration, blood samples (3 mL per bird) were centrifuged at 4°C, 1500 × g for 15 min to get the serum (0.5 mL per bird), and then, it was snap-frozen in liquid nitrogen and stored at −80°C for subsequent analysis. The weights of the body, breast muscles, and leg muscles were weighed, and the length of the jejunum was measured as well.

### Muscle index calculation

The muscle index for both breast and leg muscles was calculated using the following formula: muscle index = muscle weight (g)/body weight (g).

### Microbial genomic DNA extraction and 16S rRNA gene sequencing

Microbial community genomic DNA was extracted from the content and mucosa samples (250 mg per sample) using Fast DNA SPIN extraction kits (MP Biomedicals, Santa Ana, CA, USA) according to manufacturer’s instructions. Extracted DNA (OD 260/280 ranged from 1.8 to 2.0) was quantified using NanoDrop ND-1000 spectrophotometer (Thermo Fisher Scientific, Waltham, MA, USA) and 1% agarose gel electrophoresis, respectively. The hypervariable regions V3-V4 of the bacterial 16S rRNA gene were amplified with the forward primer 338F (5′-ACTCCTACGGGAGGCAGCA-3′) and the reverse primer 806R (5′-GGACTACHVGGGTWTCTAAT-3′). The PCR amplification of the 16S rRNA gene was performed as follows: initial denaturation at 98°C for 2 min, followed by 25 cycles of denaturation at 98°C for 15 s, annealing at 55°C for 30 s, and extension at 72°C for 30 s, with a final extension of 5 min at 72°C. The PCR mixture contains 5 × *TransStart* FastPfu buffer 4 μL, 2.5 mM dNTPs 2 μL, forward primer (5 μM) 0.8 μL, reverse primer (5 μM) 0.8 μL, *TransStart* FastPfu DNA polymerase 0.4 μL, template DNA 10 ng, and finally ddH_2_O up to 20 μL. PCR reactions were performed in triplicate. PCR amplicons were purified with Agencourt AMPure Beads (Beckman Coulter, Indianapolis, IN) and quantified using the PicoGreen dsDNA Assay Kit (Invitrogen, Carlsbad, CA, USA). The concentration of purified amplicon library of each sample was above 0.5 ng/uL. Purified amplicons were pooled in equimolar and paired-end sequenced (2 × 300 bp) on an Illumina MiSeq platform (Illumina, San Diego, USA) according to the standard protocols of Majorbio Bio-Pharm Technology Co., Ltd. (Shanghai, China), and more than 30,000 clean reads were obtained from each purified amplicon library.

### Sequencing data analysis

The raw 16S rRNA gene sequencing reads were demultiplexed, quality-filtered by Trimmomatic, and merged by fast length adjustment of short reads (FLASH). Operational taxonomic units (OTUs) with 97% similarity cutoff were clustered using UPARSE (version 7.1, http://drive5.com/uparse/), and chimeric sequences were identified and removed to get valid reads. The taxonomy of each OTU representative sequence was analyzed by ribosomal database project (RDP) Classifier (http://rdp.cme.msu.edu/) against the 16S rRNA database (Silva 138) using a confidence threshold of 0.7 [79].

To minimize the effects of sequencing depth on alpha and beta diversity measure, the reads from each sample were subsampled. The lowest valid reads of both high and low body weight samples for the jejunal content were 28,399 and for the jejunal mucosa were 17,134. Similarly, the lowest valid reads of both the FMT and the control samples for the jejunal content were 34,557 and for the jejunal mucosa were 29,970. The α-diversity was described using the Shannon index and Chao index. Principal component analysis (PCA) based on Bray-Curtis was used to estimate the dissimilarity in the community structure (β-diversity). The community composition at phylum and genus level was visualized by pie or bar chart. Linear discriminant analysis effect size (LEfSe) was performed to detect differentially abundant taxa across groups using the default parameters linear discriminant analysis (LDA > 2). Kyoto Encyclopedia of Genes and Genomes (KEGG) function annotation of the sequence was carried out based on Tax4Fun, then visualized using the statistical analysis of metagenomic profiles (STAMP) software package [80].

### Hematoxylin and eosin (H & E) staining

Breast muscles, leg muscles, and jejunum tissue samples were embedded in paraffin and then cut into 3-μm-thick sections with a rotary slicer (LEICARM2245, Leica, Germany). Slices were stained with hematoxylin and eosin following steps, which has been reported by Cui et al. [10].

### Toluidine blue staining

Toluidine blue staining was performed to observe mast cells in the jejunum. The slices of jejunum were deparaffinized twice in xylene and rehydrated in a graded series of ethanol. Then, slices were kept in toluidine blue staining solution for 5 min, rinsed with distilled water, differentiated in 95% ethanol, dehydrated in 100% ethanol, cleared in xylene, and finally mounted with coverslips [81].

### Periodic Acid-Schiff (PAS) staining

PAS staining was performed to observe goblet cells in the jejunum. Slices were deparaffinized twice in xylene and rehydrated in a graded series of ethanol. The periodic acid was added to the slides and kept for 10 min. Then, Schiff’s reagents were added onto the slides and kept in the dark for 30 min followed by flowing water washing. Finally, slides were counterstained with hematoxylin and mounted with coverslips [82].

### Immunohistochemical staining

Immunohistochemical staining was used to observe the protein expression and distribution in the jejunum following the steps described in earlier studies [10]. In brief, slices were deparaffinized twice in xylene and rehydrated in a graded series of ethanol. The antigen was repaired in sodium citrate buffer using a microwave oven and then cooled down at room temperature. For inactivation of endogenous peroxidase, 3% hydrogen peroxide (H_2_O_2_) was used, and tissues were incubated with 5% bovine serum albumin (BSA) (Boster, China) at 37°C for 30 min to block nonspecific binding sites. The primary antibody including rabbit anti-IL-1β (1:200) (WL00891, Wanleibio, China) and rabbit anti-TLR4 (1:500) (WL00196, Wanleibio, China) was used, and tissue sections were incubated at 4°C for 12 h. Then, horseradish peroxidase (HRP)-conjugated secondary antibody (Proteintech, China) was incubated for 30 min at 37°C. After DAB (Proteintech, China) staining, slices were counterstained with hematoxylin and mounted with coverslips.

### Enzyme-linked immunosorbent assay (ELISA)

Serum LPS concentration was determined using Chicken Lipopolysaccharide ELISA Kit (Jiyinmei, China) according to the manufacturer’s instructions. The absorbance was measured at 450 nm, and the average absorbance value (A450) of each sample was calculated according to the standard curve.

### Quantitative real-time polymerase chain reaction PCR (qPCR)

To compare the expression differences of related genes between groups, the total RNA was isolated from the jejunum tissue with Trizol reagent (Takara, Japan) according to the manufacturer’s instructions. Genomic DNA was removed, and 1 μg RNA of each sample was reverse transcribed into cDNA using the PrimeScript™ RT reagent Kit with gDNA Eraser (Takara, Japan). The reaction mixture (10 μL) for qPCR contained 5 μL of SYBR (Takara, Japan), 0.4 μL of forward and reverse primer, 3.2 μL of ddH_2_O, and 1 μL of template cDNA. The qPCR reactions were performed on a Bio-Rad CFX Connect real-time qPCR detection system (Bio-Rad, Hercules, CA, USA) following the steps: pre-denaturation at 95°C for 5 min, followed by 40 cycles of denaturation at 95°C for 30 s, annealing at 60°C for 30 s, and elongation at 72°C for 15 s. The primer sequences were listed in Table 1, and β-actin was chosen as a reference gene. Gene expression levels were quantified using the 2 ^−ΔΔCT^ method.

**Table 1:** List of genes and primer sequence for quantitative real-time PCR analysis

Gene	Primer sequences (5′ to 3′)	Accession No.
β-actin	f-TTGTTGACAATGGCTCCGGT	NM_205518.1
	r-TCTGGGCTTCATCACCAACG	
IL-1β	f-ACCTACAAGCTAAGTGGGCG	NM_204524.1
	r-ATACCTCCACCCCGACAAGG	
TNF-α	f-CAGATGGGAAGGGAATGAAC	AY765397.1
	r-CACACGACAGCCAAGTCAAC	
IFN-γ	f-CTCGCAACCTTCACCTCACCATC	NM_205149.1
	r-CAGGAACCAGGCACGAGCTTG	
IL-6	f-CTCCTCGCCAATCTGAAGTC	NM_204628.1
	r-AGGCACTGAAACTCCTGGTCT	
IL-12	f-ATTACTTTCCTTTGCTGCCCTTC	NM_213571.1
	r-CTGGTGTCTCATCGTTCCACTC	
TLR4	f-TGAAAGAGCTGGTGGAACCC	NM_001030693.1
	r-CCAGGACCGAGCAATGTCAA	
MyD88	f-AGGATGGTGGTCGTCATTTC	NM_001030962.2
	r-TTGGTGCAAGGATTGGTGTA	
NF-κB	f-CTACTGATTGCTGCTGGAGTTG	M86930.1
	r-CTGCTATGTGAAGAGGCGTTGT	
IL-4	f-AGCCAGCACTGCCACAAGAAC	NM_001007079.1
	r-CGTGGGACATGGTGCCTTGAG	
IL-10	f-CAGCACCAGTCATCAGCAGAGC	NM_001004414.2
	r-GCAGGTGAAGAAGCGGTGACAG	
TGF-β	f-ATGTGTTCCGCTTTAACGTGTC	NM_205454.1
	r-GCTGCTTTGCTATATGCTCATC	
MUC2	f-AATGCTGAGTTCTTGCCTAA	XM_001234581.3
	r-TGTTGCAGTTCATATCCTGGT	
Occludin	f-CGCAGATGTCCAGCGGTTACT	NM_205128.1
	r-CAGAGCAGGATGACGATGAGGAA	
ZO-1	f-CCACTGCCTACACCACCATCTC	XM_015278975.1
	r-CGTGTCACTGGGGTCCTTCAT	
Claudin-1	f-GCATGGAGGATGACCAGGTGA	NM_001013611.2
	r-GAGCCACTCTGTTGCCATACCAT	
Caspase-3	f-TCCACCGAGATACCGGACTG	NM_204725.1
	r-ACAAAACTGCTTCGCTTGCT	
Bax	f-GGGGTACGTCAATGTGGTCA	XM_015274882.1
	r-AGGAAGGCGGTGGGATAATG	
Bak	f-GTTCCGGAGCTACACCTTCT	NM_001030920.1
	r-GTACCGCTTGTTGATGTCGT	
Bcl-2	f-ATGACCGAGTACCTGAACCG	NM_205339.2
	r-CAAGAGTGATGCAAGCTCCC	
P53	f-GCTGAACCCCGACAATGAGA	NM_205264.1
	r-TTTGCAGCAGTTTCTTCCCG	

### Statistical analysis

The digital photographs were taken with a light microscope (BH-2; Olympus, Japan) using a digital camera (DP72; Olympus). There were 10 sections in each group, and 8 random visual fields of each section were selected for image acquisition according to types of the tissue. In each section, the average cross-sectional area of a single breast and leg muscle cell and positive signal of IHC was calculated with Image-Pro Plus (IPP) 6.0 software (Media Cybernetics, USA). Mast cells and goblet cells of each visual field were also counted. Analyses and graphics were obtained using Prism software 8 (GraphPad Software, Inc., San Diego, USA). All data are presented as the means ± standard error of the mean (SEM). The statistical significance of the mean values in two-group comparisons was determined using Student’s *t* test. The value of *p* < 0.05 was considered statistically significant.

## Supplementary Information

**Additional file 1: Figure S1.** Predicted function of jejunal microbiota. The third level of KEGG pathways was shown in the post-hoc plot. (A) Differential functions of jejunal microbiota in the content between high and low weight chickens. (B) Differential functions of jejunal microbiota in the mucosa between high and low weight chickens.

## Data Availability

The raw 16S rRNA gene sequencing data are available at the NCBI Sequence Read Archive (SRA), under BioProject PRJNA707106.
