# Correction: Chicken jejunal microbiota improves growth performance by mitigating intestinal inflammation

**DOI:** 10.1186/s40168-022-01330-y

**Published:** 2022-08-02

**Authors:** Xiaolong Zhang, Muhammad Akhtar, Yan Chen, Ziyu Ma, Yuyun Liang, Deshi Shi, Ranran Cheng, Lei Cui, Yafang Hu, Abdallah A. Nafady, Abdur Rahman Ansari, El-Sayed M. Abdel-Kafy, Huazhen Liu

**Affiliations:** 1 Department of Basic Veterinary Medicine, College of Animal Science and Veterinary Medicine, Huazhong Agricultural University, Wuhan, 430070 Hubei China; 2 Department of Preventive Veterinary Medicine, College of Animal Science and Veterinary Medicine, Huazhong Agricultural University, Wuhan, 430070 Hubei China; 3 Section of Anatomy and Histology, Depart- ment of Basic Sciences, College of Veterinary and Animal Sciences (CVAS) Jhang, University of Veterinary and Animal Sciences (UVAS), Lahore, Pakistan; 4 Animal Production Research Institute (APRI), Agricultural Research Center (ARC), Ministry of Agriculture, Giza, Egypt

**Correction: Microbiome 10, 107 (2022)**

**https://doi.org/10.1186/s40168-022-01299-8**

Following the publication of the original article, the author reported that an incorrect video abstract is attached to the article.

The original article [1] has been updated.
