# Supplementary figures and images for: Chicken jejunal microbiota improves growth performance by mitigating intestinal inflammation
Source: Microbiome. 2022 Jul 15;10:107. doi: 10.1186/s40168-022-01299-8 (PMC9284917; doi:10.1186/s40168-022-01299-8)

**Figure S1**
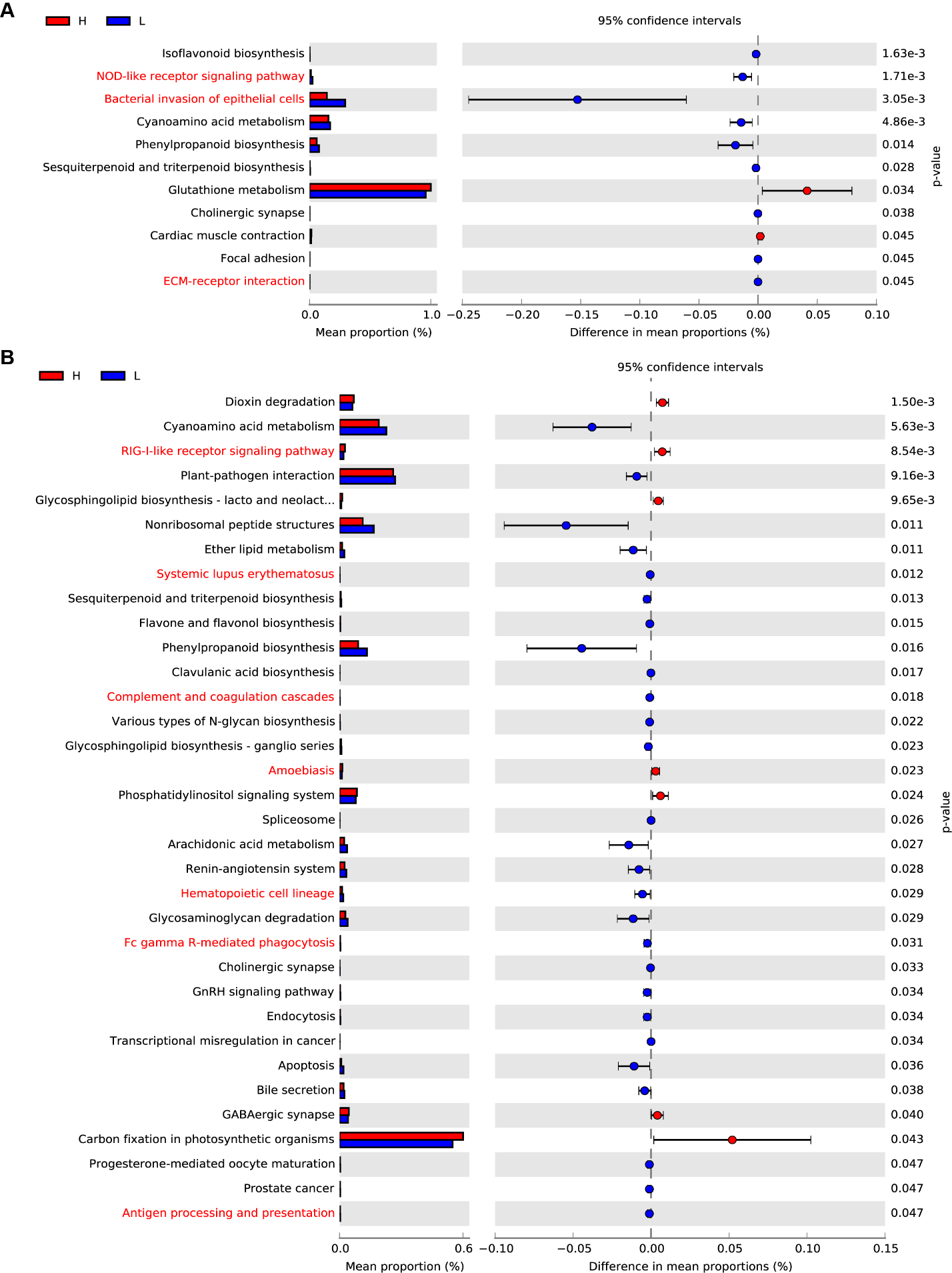

Supplement: Supplementary file 2 — Additional file 1: Figure S1. Predicted function of jejunal microbiota. The third level of KEGG pathways was shown in the post-hoc plot. (A) Differential functions of jejunal microbiota in the content between high and low weight chickens. (B) Differential functions of jejunal microbiota in the mucosa between high and low weight chickens. [file 40168_2022_1299_MOESM1_ESM.docx]
